# Non-erosive gastro-oesophageal reflux disease and incidence of oesophageal adenocarcinoma in three Nordic countries: population based cohort study

**DOI:** 10.1136/bmj-2023-076017

**Published:** 2023-09-13

**Authors:** Dag Holmberg, Giola Santoni, My von Euler-Chelpin, Martti Färkkilä, Joonas H Kauppila, John Maret-Ouda, Eivind Ness-Jensen, Jesper Lagergren

**Affiliations:** 1 Department of Molecular Medicine and Surgery, Karolinska Institutet and Karolinska University Hospital, Stockholm, Sweden; 2 Department of Public Health, University of Copenhagen, Copenhagen, Denmark; 3 Clinic of Gastroenterology, University of Helsinki and Helsinki University Hospital, Helsinki, Finland; 4 Department of Surgery, Oulu University Hospital and University of Oulu, Oulu, Finland; 5 Centre for Clinical Research Sörmland, Uppsala University, Eskilstuna, Sweden; 6 Department of Medical Epidemiology and Biostatistics, Karolinska Institutet, Stockholm, Sweden; 7 Department of Public Health and Nursing, NTNU, Norwegian University of Science and Technology, Trondheim/Levanger, Norway; 8 Medical Department, Levanger Hospital, Nord-Trøndelag Hospital Trust, Levanger, Norway; 9 School of Cancer and Pharmaceutical Sciences, King’s College London, London, UK

## Abstract

**Objective:**

To assess the incidence rate of oesophageal adenocarcinoma among patients with non-erosive gastro-oesophageal reflux disease compared with the general population.

**Design:**

Population based cohort study.

**Setting:**

All patients in hospital and specialised outpatient healthcare in Denmark, Finland, and Sweden from 1 January 1987 to 31 December 2019.

**Participants:**

486 556 adults (>18 years) who underwent endoscopy were eligible for inclusion: 285 811 patients were included in the non-erosive gastro-oesophageal reflux disease cohort and 200 745 patients in the validation cohort with erosive gastro-oesophageal reflux disease.

**Exposures:**

Non-erosive gastro-oesophageal reflux disease was defined by an absence of oesophagitis and any other oesophageal diagnosis at endoscopy. Erosive gastro-oesophageal reflux disease was examined for comparison reasons and was defined by the presence of oesophagitis at endoscopy.

**Main outcome measures:**

The incidence rate of oesophageal adenocarcinoma was assessed for up to 31 years of follow-up. Standardised incidence ratios with 95% confidence intervals were calculated by dividing the observed number of oesophageal adenocarcinomas in each of the gastro-oesophageal reflux disease cohorts by the expected number, derived from the general populations in Denmark, Finland, and Sweden of the corresponding age, sex, and calendar period.

**Results:**

Among 285 811 patients with non-erosive gastro-oesophageal reflux disease, 228 developed oesophageal adenocarcinomas during 2 081 051 person-years of follow-up. The incidence rate of oesophageal adenocarcinoma in patients with non-erosive gastro-oesophageal reflux disease was 11.0/100 000 person-years. The incidence was similar to that of the general population (standardised incidence ratio 1.04 (95% confidence interval 0.91 to 1.18)), and did not increase with longer follow-up (1.07 (0.65 to 1.65) for 15-31 years of follow-up). For validity reasons, we also analysed people with erosive oesophagitis at endoscopy (200 745 patients, 1 750 249 person-years, and 542 oesophageal adenocarcinomas, corresponding to an incidence rate of 31.0/100 000 person-years) showing an increased overall standardised incidence ratio of oesophageal adenocarcinoma (2.36 (2.17 to 2.57)), which became more pronounced with longer follow-up.

**Conclusions:**

Patients with non-erosive gastro-oesophageal reflux disease seem to have a similar incidence of oesophageal adenocarcinoma as the general population. This finding suggests that endoscopically confirmed non-erosive gastro-oesophageal reflux disease does not require additional endoscopic monitoring for oesophageal adenocarcinoma.

## Introduction

Gastro-oesophageal reflux disease (GORD) is defined by at least weekly symptoms of troublesome heartburn or regurgitation or complications related to GORD,^1^ which occurs in approximately 20% of adults in high income countries.^2^ ^3^ ^4^ GORD is the main risk factor for oesophageal adenocarcinoma, a tumour with increasing incidence and poor survival.^5^ ^6^ ^7^ ^8^ ^9^ ^10^ Patients with GORD symptoms are frequently referred for upper endoscopy to search for mucosal abnormalities, including erosive oesophagitis and metaplasia (Barrett’s oesophagus), the precursor conditions to oesophageal adenocarcinoma. However, the most common finding at an upper endoscopy examination for GORD symptoms is a normal-appearing oesophageal mucosa, representing non-erosive GORD.^1^ ^11^ ^12^ The association between oesophagitis and oesophageal adenocarcinoma is well established, but no previous study has been able to reliably examine the risk of developing oesophageal adenocarcinoma among patients with endoscopically confirmed non-erosive GORD. This research requires a large cohort of patients with GORD having undergone a normal upper endoscopy with an extensive follow-up.

We aimed to provide an answer to whether non-erosive GORD is associated with oesophageal adenocarcinoma by examining a large and unselected cohort with a long and complete follow-up, retrieved from three Nordic countries.

## Methods

### Design

In this population based cohort study, we encompassed all healthcare in Denmark, Finland, and Sweden. The total study period was from 1 January 1987 to 31 December 2019, but the exact years of study differed between the participating countries (1995-2019 in Denmark, 1987-18 in Finland, and 2006-19 in Sweden). Approvals were obtained from the appropriate ethical review boards, data inspectorates, and government authorities in the three countries.

### Source cohort

The source cohort included all patients diagnosed and recorded with GORD in hospital or specialised outpatient healthcare by a physician in any of the three participating Nordic countries who underwent at least one upper endoscopy. For each of these patients, the cohort holds all data from nationwide complete patient registries, cancer registries, and cause of death registries. All healthcare institutions are required by law to report continuously and prospectively all collected data regarding admissions, discharges, diagnoses, and procedures in hospital and specialised outpatient healthcare, and causes of death to the registry holders. These data include the diagnostic codes for GORD according to the International Classification of Diseases and procedural codes for upper gastrointestinal endoscopy according to the Nordic Medico-Statistical Committee Classification of Surgical Procedures and country specific procedure classifications (appendix). All diagnostic and procedural codes are determined and documented by physicians. Personal identity numbers are used by all residents and recorded in the registries; these numbers enabled accurate linkages and merging of information for each participant. The variables recorded in the national healthcare registries have been validated for accuracy and completeness with excellent results.^13^ ^14^ ^15^ ^16^ ^17^ ^18^ ^19^ ^20^ Specifically for this study, the completeness and validity are particularly high for diagnoses linked to procedures, such as upper endoscopy,^14^ and for oesophageal adenocarcinoma diagnosis (>98% completeness).^17^

### Non-erosive GORD cohort

All patients were eligible for inclusion in the non-erosive GORD cohort if they had a GORD diagnosis, based on GORD symptoms, and a normal finding from their first upper endoscopy examination. Patients were strongly advised not to take antireflux medication for a few weeks before a diagnostic upper endoscopy to avoid misclassification of erosive GORD. A normal endoscopy finding was defined as no erosive oesophagitis or other oesophageal diagnosis from the date of endoscopy until 12 months later. The oesophageal diagnoses were identified by codes according to the International Classification of Diseases (ICD) versions 9 and 10 (appendix). The 12 month post-endoscopy observation time was used because oesophageal diagnoses might not always be apparent on a first upper endoscopy examination or need histological confirmation. This time period allowed for additional diagnostic workup, including repeated endoscopies and final histological diagnosis. To avoid immortal time bias, follow-up started after this 12 month period for all patients, and those who developed any of the study endpoints during this 12 month period were excluded from the study. Additionally, we excluded patients with any upper endoscopy conducted before the study period, any diagnosis of oesophageal cancer, gastric cancer, or Barrett’s oesophagus at baseline, and any first upper endoscopy at younger than 18 years of age or older than 90 years.

### Erosive GORD cohort for validation

To validate and contrast the findings observed in the non-erosive GORD cohort, we included all patients in the source cohort diagnosed with erosive GORD (ie, oesophagitis), which is a well established risk factor for oesophageal adenocarcinoma. Similar to the non-erosive GORD cohort, patients were required to be without any other oesophageal diagnosis within 12 months of the first upper endoscopy (appendix) and the follow-up commenced thereafter. The same exclusion criteria as those described for the non-erosive GORD cohort were also made for this cohort.

### Outcome

The outcome was incidence of oesophageal adenocarcinoma, defined by the ICD version 7, codes 150 (oesophagus) or 1511 (gastric cardia), combined with the WHO/HS/CANC/24.1 histology code 096 (adenocarcinoma) or ICD-10, codes C15 (oesophagus) or C160 (cardia), combined with the histology code ICD-O/3 8140-8149, 8160-8162, 8190-8221, 8260-8337, 8350-8551, 8570-8576, 8940-8941 (all with 3 as fifth digit) (adenocarcinoma). All codes were as identified through the national cancer registries in the three countries.

### Statistical analysis

Standardised incidence ratios with 95% confidence intervals were calculated by dividing the observed numbers of oesophageal adenocarcinoma cases separately among patients in the non-erosive GORD cohort and the erosive GORD cohort by the expected numbers. The expected numbers were calculated by multiplying the observed person-time by the demographic variables age (in five year groups), sex (men and women), and calendar year (in five year categories). For this calculation, we used incidence rates in the general populations of Denmark, Finland, and Sweden according to data from the national cancer registries separately for the non-erosive GORD cohort and the erosive GORD cohort. The main analyses assessed changes in standardised incidence ratios across five periods of follow-up: <1 year, 1-4 years, 5-9 years, 10-14 years, and 15-31 years. Changes in standardised incidence ratios over time were plotted using Poisson regression, where follow-up time was modelled with restricted cubic splines of time with three knots (the chosen knots gave the model with the smallest Akaike Information Criterion). Stratified analyses calculated standardised incidence ratios for subgroups of the demographic variables of age (two groups divided by the median), sex (men and women), and calendar period (two groups divided by the median calendar year).

The follow-up started 12 months after the index endoscopy and ended on oesophageal cancer diagnosis, death, or the end of the study period, whichever occurred first. The personal identity numbers combined with the inclusion of all healthcare pertaining to the residents of the included countries that was captured through national registries meant that follow-up was complete. All data management and statistical analyses were performed in 2022-23 by an experienced biostatistician (GS) who followed a detailed preplanned study protocol and used the statistical software Stata (version 16, StataCorp, College Station, TX).

### Patient and public involvement statement

We co-organise a patient group consisting of 8-12 patients having undergone treatment for oesophageal or gastric cancer. We have discussed this study with the patient group and they have given it their full support, but they have not changed anything in the original study plan.

## Results

### Patients

Among all 486 556 patients who underwent endoscopy for GORD, the non-erosive GORD cohort included 285 811 patients, and the validation cohort with erosive GORD cohort consisted of 200 745 patients (table 1). The non-erosive GORD cohort contained more women (58.7%) compared with the erosive GORD cohort (44.6% women), but the cohorts were otherwise similar (table 1). 

**Table 1:** Characteristics of patients with non-erosive and erosive gastro-oesophageal reflux disease (GORD)

**Characteristics**	**Non-erosive GORD cohort, no. (%) (n=285 811)**	**Erosive GORD cohort, no. (%) (n=200 745)**
Follow-up (years):		
Maximum	31	31
Median (interquartile range)	6.3 (3.0-10.4)	7.8 (3.8-12.3)
Age (years):		
Median (interquartile range)	59 (44-70)	58 (45-69)
Sex, no. (%):		
Men	118 061 (41.3)	111 285 (55.4)
Women	167 750 (58.7)	89 460 (44.6)
Calendar year:		
Median (interquartile range)	2012 (2008-2016)	2010 (2005-2014)
Country, no. (%):		
Denmark	79 195 (27.7)	78 858 (39.3)
Finland	69 671 (24.4)	49 835 (24.8)
Sweden	136 945 (47.9)	72 052 (35.9)
Oesophageal adenocarcinoma	228 (0.08)	542 (0.27)
Time to diagnosis (years):		
Median (interquartile range)	6.2 (3.0-9.6)	7.0 (3.3-11.9)

### Incidence of oesophageal adenocarcinoma

**Fig 1:**
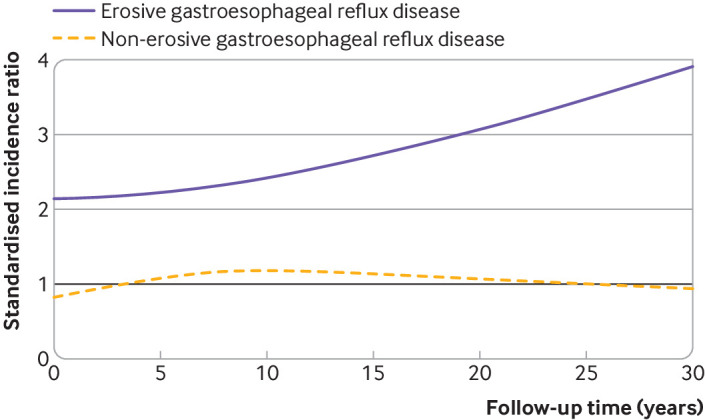
Standardised incidence ratios of oesophageal adenocarcinoma over follow-up time among patients with non-erosive (dashed line) and erosive (solid line) gastro-oesophageal reflux disease compared with the general population of the same age, sex, and calendar period

During follow-up of 2 081 051 person-years (median follow-up time 6.3 years) in the non-erosive GORD cohort, 228 (0.08%) patients developed oesophageal adenocarcinoma, 60 499 (21.2%) underwent follow-up endoscopy, and 3039 (1.1%) underwent antireflux surgery. The overall incidence rate of oesophageal adenocarcinoma was 11.0 cases per 100 000 person-years. The overall standardised incidence ratio of oesophageal adenocarcinoma was 1.04 (95% confidence interval 0.91 to 1.18). The standardised incidence ratios showed no increasing trend during the maximum of 31 years of follow-up, and the standardised incidence ratio for 15-31 years of follow-up was 1.07 (0.65 to 1.65) (table 2, fig 1). Women retained a slightly increased standardised incidence ratio of oesophageal adenocarcinoma (1.38 (1.08 to 1.73)), but otherwise, no major differences were noted in standardised incidence ratios between patients of different ages or calendar periods (table 2).

**Table 2:** Standardised incidence ratio of oesophageal adenocarcinoma among patients with non-erosive gastro-oesophageal reflux disease compared with the corresponding general population

Characteristics	Person-years	Oesophageal adenocarcinomas	Standardised incidence ratio (95% confidence interval)
Follow-up:			
0-31 years	2 081 051	228	1.04 (0.91 to 1.18)
<1 year	273 787	23	0.97 (0.61 to 1.45)
1-4 years	861 035	69	0.86 (0.67 to 1.09)
5-9 years	608 141	82	1.26 (1.00 to 1.56)
10-14 years	232 984	34	1.09 (0.75 to 1.52)
15-31 years	105 105	20	1.07 (0.65 to 1.65)
Age (years):			
<60	1 231 438	74	1.11 (0.87 to 1.39)
≥60	849 613	154	1.01 (0.86 to 1.18)
Sex:			
Men	866 646	154	0.93 (0.79 to 1.09)
Women	1 214 405	74	1.38 (1.08 to 1.73)
Calendar period:			
1987-2010	1 238 536	161	1.16 (0.99 to 1.36)
2010-2018	842 515	67	0.83 (0.64 to 1.06)

For validation, we followed up 1 750 249 person-years (median follow-up time 7.8 years) in the erosive GORD cohort, which identified 542 (0.27%) patients who developed oesophageal adenocarcinoma, 54 001 (26.9%) who underwent follow-up endoscopy, and 3704 (1.9%) who underwent antireflux surgery. The incidence rate of oesophageal adenocarcinoma was 31.0 cases per 100 000 person-years. The overall standardised incidence ratio was 2.36 (95% confidence interval 2.17 to 2.57), and the point estimates increased from years one to four and gradually for each follow-up period later, with a standardised incidence ratio 2.73 (2.15 to 3.42) for the 15-31 years of follow-up category (table 3, fig 1). The standardised incidence ratios were increased in all analyses stratified by age, sex, and calendar year (table 3).

**Table 3:** Standardised incidence ratio of oesophageal adenocarcinoma among patients with erosive gastro-oesophageal reflux disease compared with the corresponding general population

Characteristics	Person-years	Oesophageal adenocarcinomas	Standardised incidence ratio (95% confidence interval)
Follow-up (years):			
0-31	1 750 249	542	2.36 (2.17 to 2.57)
<1	193 684	47	2.32 (1.70 to 3.08)
1-4	624 114	155	2.14 (1.82 to 2.51)
5-9	522 687	156	2.28 (1.94 to 2.67)
10-14	254 708	108	2.65 (2.17 to 3.20)
15-31	137 056	76	2.73 (2.15 to 3.42)
Age (years):			
<60	1 111 935	225	2.63 (2.30 to 3.00)
≥60	638 314	317	2.20 (1.97 to 2.46)
Sex:			
Men	967 193	435	2.27 (2.06 to 2.49)
Women	783 056	107	2.82 (2.31 to 3.41)
Calendar period:			
1987-2009	1 257 161	437	2.57 (2.34 to 2.82)
2010-2018	493 088	105	1.76 (1.44 to 2.13)

## Discussion

### Principal findings

This study found no increased incidence of oesophageal adenocarcinoma in patients with non-erosive GORD, even after a long observation time, compared with the corresponding general population. Patients with erosive GORD at endoscopy had, as expected, a notable increased incidence of this tumour.

### Strengths and limitations of the study

This study shows that patients with GORD but no oesophageal mucosal abnormalities have a similar incidence of oesophageal adenocarcinoma as the general population. Among methodological strengths are the population based design, the large sample size, and the long follow-up. We merged data from national health registries in Denmark, Finland, and Sweden, which have been validated for excellent completeness and quality for the variables used in this study. The personal identification number system and the complete national registries prevented losses to follow-up. The association between erosive GORD and oesophageal adenocarcinoma adds validity to the results for the non-erosive GORD cohort. A limitation of the observational design is the risk of residual or unknown confounding. However, the established risk factors for oesophageal adenocarcinoma other than GORD (ie, obesity and to a lesser degree tobacco smoking) are more common in patients with GORD than in the general population and should lead to an unadjusted increase in standardised incidence ratios in the patients with non-erosive GORD rather than decrease them. Similarly, the higher propensity to repeat endoscopy in patients with GORD could lead to earlier diagnosis in asymptomatic oesophageal adenocarcinoma, but such detection bias would artificially increase standardised incidence ratios in the non-erosive GORD cohort. Patients with warning symptoms of symptomatic oesophageal adenocarcinoma (eg, dysphagia) are rapidly evaluated and access to endoscopy should be similar between the groups without major bias in detection. Thus, differences in endoscopy access between the non-erosive GORD cohort and the general population should not contribute to the null finding. The general population contained patients with GORD, which dilutes the associations. Nevertheless, such dilution should be minimal because these cohorts represented a small number of the population in these countries and should not explain the absence of association between non-erosive GORD and oesophageal adenocarcinoma found in this study. The general population may represent the best option for comparison because it makes up a large, unselected and stable comparison cohort. Information about severity of GORD symptoms were unavailable and is unfeasible to collect for a study of the size and design required. Exclusion of all patients with differential diagnoses that may be mixed up with GORD was not possible, and this occurrence might be more frequent in the non-erosive GORD cohort. However, all patients had a GORD diagnosis established by a physician, which reduced misclassification.

### Comparison with other studies

One previous study, a Danish population based cohort study consisting of 7655 patients, has attempted to assess the risk of oesophageal adenocarcinoma specifically in patients with non-erosive GORD.^21^ Only one patient developed oesophageal adenocarcinoma during follow-up, prohibiting conclusive results, but the study still suggested a low incidence of oesophageal adenocarcinoma in patients with non-erosive GORD.^21^ This low incidence has found some indirect support in smaller natural history studies showing that non-erosive GORD seldom progresses to erosive GORD or Barrett’s oesophagus.^12^ ^22^ Thus, non-erosive and erosive GORD have been forwarded as different phenotypes, where patients seldom advance from non-erosive to erosive GORD.^23^

### Interpretation of the study findings

In the non-erosive cohort, the incidence of oesophageal adenocarcinoma was similar to that of the corresponding general population without any increasing trend for up to 31 years of follow-up, while the erosive GORD cohort showed continuously increased standardised incidence ratios over time. These striking differences in trends strengthen the evidence for no association between confirmed non-erosive GORD and oesophageal adenocarcinoma. Although not statistically significant, the point estimate of oesophageal adenocarcinoma was decreased within the first one to four years of follow-up. This suggests little influence of endoscopy screening for oesophageal adenocarcinoma. Women were more likely to present with non-erosive GORD than men, which is in line with the literature,^24^ but the finding that they retained a slightly increased incidence of developing oesophageal adenocarcinoma was less expected. The increase was moderate and might be explained by random error, but should be assessed in future research.

### Clinical implications

The findings from this study may show a distinctive difference in how to manage oesophageal adenocarcinoma risk in patients with GORD. Patients with GORD symptoms, but with normal oesophageal mucosa, seem to be largely unrelated to oesophageal adenocarcinoma, even after a long follow-up. The finding may represent a shift in how to consider patients’ risk of tumour development based on endoscopic GORD phenotype, where patients with non-erosive GORD may be treated as the general population, whereas those with erosive GORD might benefit from being re-assessed.

General practitioners and various specialists may see many patients with recurring or continuous reflux symptoms requiring medication. These patients often undergo upper endoscopy with the main purpose of excluding premalignant and malignant lesions. In this study, a fifth of all patients with non-erosive GORD underwent at least one repeated upper endoscopy during the study period, but the yield of oesophageal adenocarcinoma from these repeated examinations was low. This study reflected routine healthcare, and patients who underwent repeated endoscopy likely had more persistent, severe, or new symptoms, and represents a selected subpopulation. Therefore, reliably assessing the incidence of intermediate steps to oesophageal adenocarcinoma (eg, erosive oesophagitis or Barrett’s oesophagus) was not possible. However, the null association between non-erosive GORD and oesophageal adenocarcinoma suggests that the transition from non-erosive GORD to oesophagitis and Barrett’s oesophagus is rare, given the strong association between these conditions and oesophageal adenocarcinoma. Oesophageal adenocarcinoma is the final stage of the disease spectrum and the most important outcome to study. Thus, this study suggests that physicians do not need to consider referring patients with GORD with a previous normal upper endoscopy for repeat endoscopy unless they develop warning symptoms of oesophageal adenocarcinoma, mainly dysphagia, as recommended for all individuals. This message contrasts with today’s clinical practice, in which many patients with diagnosed non-erosive GORD undergo repeated upper endoscopies, which might be both costly and ineffective.^25^

### Conclusions

This three country, population based cohort study with long and complete follow-up noted that patients with GORD symptoms with a normal upper endoscopy (ie, non-erosive GORD), had no increased incidence of oesophageal adenocarcinoma compared with the corresponding general population. This finding suggests that patients with confirmed non-erosive GORD are not susceptible to develop oesophageal adenocarcinoma and may not require repeated endoscopic examinations regarding assessment of cancer risk.

**What is already known on this topic**

Gastro-oesophageal reflux disease is a major risk factor of oesophageal adenocarcinoma
Whether individuals with non-erosive gastro-oesophageal reflux disease (ie, typical symptoms combined with a normal upper endoscopy) are at increased risk of oesophageal adenocarcinoma is unknown

**What this study adds**

Patients with non-erosive gastro-oesophageal reflux disease are at similar risk of oesophageal adenocarcinoma as the corresponding general population
Patients with confirmed non-erosive gastroesophageal reflux disease do not require additional endoscopic monitoring for oesophageal adenocarcinoma

## Data Availability

Data may be obtained from a third party and are not publicly available. Protocols and statistical analysis plans will be shared on reasonable request to the corresponding author.

## Web extra

Extra material supplied by authors

Web appendix: Online appendix

## Notes

Contributors: DH, GS, and JL conceived the idea of the study. JL provided funding and supervision of the study. DH, MvE-C, and JHK acquired data. All authors designed the study. GS curated and analysed data, and the results were interpreted by the DH, GS, and JL. DH, GS, and JL wrote the original manuscript draft, which was reviewed and edited by all authors. The corresponding author attests that all listed authors meet authorship criteria and that no others meeting the criteria have been omitted. DH is the guarantor.

Patient consent: No individual level data are provided in this study.

The lead author (the manuscript’s guarantor) affirms that the manuscript is an honest, accurate, and transparent account of the study being reported; that no important aspects of the study have been omitted; and that any discrepancies from the study as planned (and, if relevant, registered) have been explained.

Dissemination to participants and related patient and public communities: The study findings will be disseminated through patient organisations, national healthcare programs, and organisations that keep the relevant international clinical guidelines.

Provenance and peer review: Not commissioned; externally peer reviewed.
