# Supplementary material for: Non-erosive gastro-oesophageal reflux disease and incidence of oesophageal adenocarcinoma in three Nordic countries: population based cohort study
Source: BMJ. 2023 Sep 13;382:e076017. doi: 10.1136/bmj-2023-076017 (PMC10496574; doi:10.1136/bmj-2023-076017)
Supplement: Supplementary file 1 — Web appendix: Online appendix [file hold076017.ww.pdf]

Appendix

**Table 1. Definition of GERD in the Nordic countries.**	
**ICD version**	**ICD codes***
**ICD-9 (FIN)**	7871A; 5513A; 5301A-D; 5301X
**ICD-9 (INT)**	787B; 553D; 530B-C
**ICD-10**	K20; K21; K22.7; K44; R12
***Danish ICD-10 codes start with the letter "D"; otherwise they are identical to standard ICD-10**	

**Table 2. Definition of endoscopy in the Nordic countries.**					
	**Operation classification <1997**			**Operation classification ≥1997**	
	**Denmark: Operations- og Behandlingsklassifikation, Sundhedstyrelsen 1988**	**Finland: Toimenpidenimikkeistö 1983**	**Sweden: Klassifikation av operationer**	**NOMESCO***	**NOMESCO (Fi)**
**Gastroscopy with/without biopsy**	9101	1300; 1310	4480; 9004	UJD02; UJD05	UJD02; UJD10
***Danish NOMESCO codes start with the letter "K"; otherwise they are identical to standard NOMESCO**					

**Table 3. Definition of esophageal and gastric cancer in the Nordic countries.**								
	**ICD-8 (DK)**	**ICD-8 (SWE)**	**ICD-9 (FIN)**	**ICD-9 (SWE)**	**ICD-9 (INT)**	**ICD-10**	**ICD-7**	**ICDO3**
**Esophageal cancer**	150; 15109	150; 15101	150; 1510 (A-X)	150; 1510	150; 1510	C15; C160	150; 1511	C15; C160
**Gastric cancer**	15119; 15180; 15181; 15189; 15199	15111, 15181, 15187, 15199	1511-1519 (A-X)	1511; 1513; 1514; 1518; 1519	1511; 1512; 1513; 1514; 1515; 1516; 1518; 1519; 20963	C161; C162; C163; C164; C165; C166; C168; C169.	1510; 1519;1518	C161; C162; C163; C164; C165; C166; C169

**Table 4. Definition of esophagitis, Barrett's esophagus, and diseases of the esophagus in the Nordic countries.**		
	**ICD-9 (FIN)**	**ICD-10 (INT)**
**Erosive esophagitis**		K21.0
**Esophagitis**	5301A	K20; K21.0
**Barrett's esophagus**	5501B	K227
**Other diseases of the esophagus**	5300A; 5302A-B; 5303A-B; 5304; 5305A-B; 5306A-X; 5307A; 5309X	K220-K226; K228-K229
*Danish ICD-10 codes start with the letter "D"; otherwise they are identical to standard ICD-10		
